# Accuracy of low-weight versus standard syringe infusion pump devices depending on altitude

**DOI:** 10.1186/s13049-019-0643-1

**Published:** 2019-07-11

**Authors:** Marc Blancher, Maxence Repellin, Maxime Maignan, Cyrielle Clapé, Arnaud Perrin, José Labarère, Guillaume Debaty, Damien Viglino

**Affiliations:** 1 Grenoble-Alps University - Emergency Department and Mobile Intensive Care Unit, Grenoble-Alps University Hospital, Grenoble, France; 2 INSERM U1042, HP2 Laboratory, Grenoble-Alps University, Grenoble, France; 3 Quality of Care Unit, Grenoble-Alps University Hospital, Grenoble, France; 4 Grenoble-Alps University, CNRS UMR 5525, TIMC-IMAG Laboratory, PRETA Team, Grenoble, France

**Keywords:** Syringe infusion pump, Emergency medicine, Intensive care unit, Mountain rescue, Vasoactive drugs, Emergency helicopter, Altitude, Air med

## Abstract

**Background:**

Intravenous drug infusions in critically ill patients require accurate syringe infusion pumps (SIPs). This is particularly important during transportation of critically ill patients by helicopter emergency medical services (HEMS), where altitude may influence device performance. Because weight is a real concern in HEMS, new low-weight devices are very appealing.

The aim of this study was to compare infusion flow rates delivered by low-weight versus standard SIP devices, in the prehospital emergency medicine setting, at different altitudes.

**Methods:**

We conducted a comparative bench study involving five SIP devices (two standard and three low-weight models) at 300, 1700 and 3000 m altitude. The primary endpoint was the flow rate delivered by SIPs for prespecified values. We used two methods to measure flow. The normative method consisted in measuring weight (method A) and the alternate method consisted in measuring instantaneous flow (method B).

**Results:**

Using method A, no significant differences were found in median flow rates and interquartile range depending on device and altitude for a prespecified 10-mL/h flow. However, method B showed that low-weight SIPs delivered multiple sequential boluses with substantial variations (1.2–15.8 mL/h) rather than a prespecified continuous 5-mL/h flow. At 1700 m altitude, the interquartile range of delivered flows increased only for low-weight devices (*p* for interaction< 0.001).

**Conclusions:**

Despite satisfactory normative tests, low-weight SIPs deliver discontinuous flow with potential clinical implications for critically ill patients receiving vasoactive drugs. This study also highlights a thus far unknown negative impact of altitude on SIP function. We believe that normative requirements for SIP approval should be revised accordingly.

## Background

Transporting critically ill patients by the air may greatly benefit patients by increasing rapid access to an adapted medical facility [1]. Even though mortality may increase during transportation, using adapted human resources and medical equipment such as syringe infusion pumps (SIPs) can minimize risks [2]. These electrical medical devices provide constant flow of drug infusion. They contribute to patient safety, avoiding mistakes during administration of multiple drugs, particularly in critical care patients. [3]. They are required for vasoactive drug infusion (such as adrenaline or noradrenaline). Indeed, when using drugs with a narrow safety margin, slight changes in administrated volumes may lead to substantial hemodynamic changes. This has been observed during syringe changeover [4, 5], and accurate SIPs participate to improve the safety of the medication process [6, 7].

These devices have undergone high-level technological development. They allow constant delivery of flow in intravenous circulation by adapting the pushing power delivered into the syringe to the flow resistance in infusion lines (adapting the type of syringes, extension lines, and/or catheters) and to intravascular pressure prevailing at the injection site. They are part of the usual medical equipment for prehospital medical management of patients in a critical condition, in countries with physician-staffed services using ground ambulances, air ambulances or helicopter emergency medical services (HEMS), and for those with medical mountain rescue services. However, these devices must be adapted to the some extreme conditions related to the prehospital transportation, whenever possible meeting specific needs in terms of low weight, size and resistance.

A low-weight SIP model (0.22 kg), Micropump™ (Micrel, Greece), which is ten times lighter than standard models, has been developed. Its low weight is highly advantageous, particularly for mountain rescue and HEMS. As for any electrical medical equipment, this device complies with the IEC 60601-2-24 [8] standard, which is necessary to obtain European CE Marking or US Food and Drug Administration approval. It was manufactured using mechanical technology only, while standard models use both electronic and mechanic technologies.

The aim of this study was to compare infusion flow delivered by low-weight versus standard SIP models, at different altitudes. We hypothesized that the flow delivered by SIPs differed depending on the model and altitude.

## Methods

### Study design

**Fig. 1:**
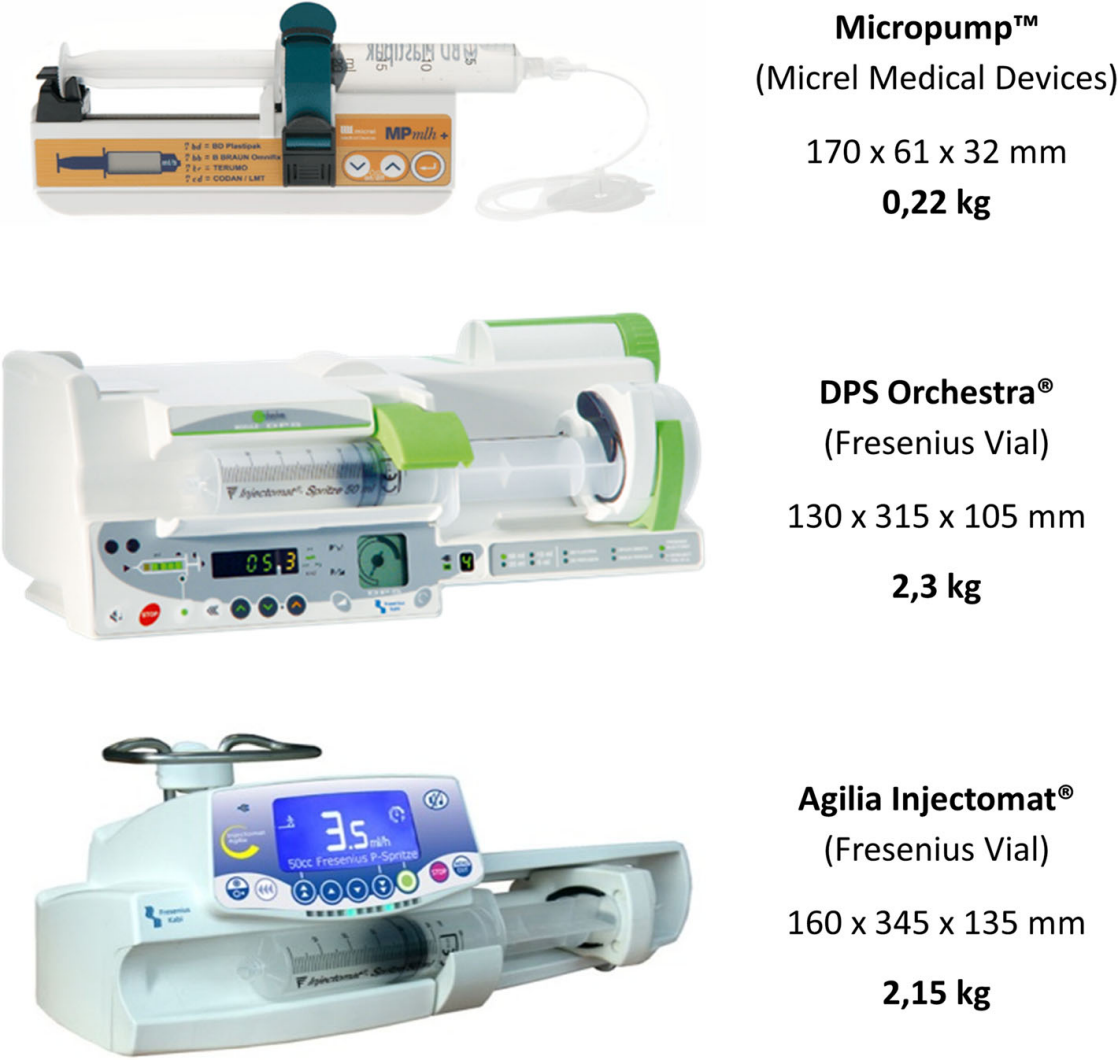
Syringe infusion pumps

We conducted an experimental, prospective and comparative bench study from February to April 2015 at Grenoble-Alps University Hospital (altitude 300 m), at the mountain rescue medical basecamp of l’Alpe d’Huez at 1700 m altitude and at the top of l’Alpe d’Huez ski resort (altitude 3000 m). No institutional review board approval was required because neither patients nor volunteers were involved. Three Micropump models (“Micropump™ MPmlh + multi-syringes” from Micrel Medical Devices S.A., Gerakas, Greece), one Agilia Injectomat® (Fresenius Vial S.A.S, Brézins, France), and one DPS Orchestra® (Fresenius Vial) were tested (Fig. 1). Each device was previously controlled and considered as ready for use by the maintenance department at our institution.

### Measurement protocol

Two different measurement methods were performed during a 2-h period of infusion.

### Method A

Weight method. This protocol, which is used by manufacturers, complied with the IEC 60601-2-24 standard [8] for electrical medical devices. The range of variance, as defined by manufacturers, was ±3% of the target infusion flow rate during the 2nd h for the Fresenius® devices and ± 4% for the Micrel® devices. SIPs were tested with an infusion flow rate set at 10 mL/h at 300 m and 1700 m at 22 °C (71.6 °F). Every 5 min for 2 h, delivered volumes of demineralized water were collected in 10-mL capacity tubes (24 measurements). The tubes were weighed beforehand, with a high-precision scale (Sartorius ME235S; Sartorius Stedim Biotech®; Aubagne, France) in the Institute of Biology and Pathology of the Grenoble-Alps University Hospital. When filled, the tubes were immediately closed to prevent evaporation. They were weighed again to calculate the volume delivered every 5 min. Volume was determined considering the difference between final weight and initial weight, for a density of 1 mL/g. The flow rate (FR) in millilitres per hour (mL/h) was calculated as follows: FR (mL/h) = volume (mL) × 60 / 5. The same measurement protocol was used for a flow rate set at 5 mL/h at 3000 m altitude at 22 °C.

### Method B

#### Automatic flow measurement

This method consisted in measuring instantaneous delivered fluid flow. We used an automatic flow measurement device: CORI-FLOW™ flow meter (Bronkhorst®, Ruurlo, the Netherlands), which measures instantaneous flow rates (100 measures per second). It also calculates overshoot and possible boluses over the range. We took measurements over 2 h for two Micrel Micropump™ models and the two standard models (Agilia Injectomat® and DPS Orchestra®), with an infusion flow rate set at 5 mL/h, at 300 m and 1700 m, at 22 °C (71.6 °F). A sample of 1200 measurements was extracted during the 2nd h of infusion at steady state.

For both methods, the test bed consisted of a half-filled 50-mL syringe (BD Plastipak, Becton, Dickinson and Company (BD)®, Franckin Lakes, NJ, USA), positioned in the SIP; an infusion line: Injectomat Line (Fresenius Vial S.A.S, Brézins, France) connected to an 18-G catheter at the same height as the syringe’s outlet.

For method A (weight method), catheters were placed directly in the measuring tubes. For method B (automatic flow), the circuit crossed the flow meter.

### Data collection

For method A, the volumes delivered were weighed with a precision scale and entered into a Microsoft Excel spreadsheet (Microsoft*®, Albuquerque, New Mexico, USA)*.

For method B, flow, starting time (defined as the time to reach the speed of 4.75 mL/h); cycle time (defined as the time between the beginning of a cycle and the beginning of the next one), representing the mean time between two modifications (increase/decrease/stop/start) of the flow detected by the flowmeter; maximum flow value exceeding the target set (expressed as a percentage of the target set) and calculated bolus volume (defined as the maximum overshoot “exceeding the set” during the infusion) were collected and processed using MATLAB® v R2014b software (MathWorks®, Natick, MA, USA).

### Statistical a1nalysis

Continuous variables with highly skewed distributions were reported as median along with 25th–75th percentiles and range (i.e., lowest and highest values). We performed median (i.e., 50th percentiles) and interquartile (i.e., difference between 75th and 25th percentiles) regressions to examine whether median flow infusion rates and interquartile range differed depending on SIP device and altitude (300 versus 1700 m), respectively. A first-order interaction term was tested for statistical significance to examine whether the accuracy of the devices varied for the different altitudes.

Two-sided *P*-values less than 0.05 were considered statistically significant. All analyses were performed using Stata SE version 14.0 (Stata Corporation, College Station, TX, USA).

## Results

For prespecified flow rate set at 10 mL/h (Table 1), the weight measurement method (method A) did not show any significant differences in median infusion flow rate and interquartile range depending on the device (*p* = 0.83 and *p* = 0.27, respectively) and altitude (*p* = 0.15 and *p* = 0.71, respectively). No significant first-order interaction was observed between devices and altitude levels for median flow rates (*p* = 0.32) and interquartile range (*p* = 0.66). There was no difference between devices when comparing median flow and interquartile range at 300 m (*p* = 0.25) or 1700 m (*p* = 0.16), as shown in Table 1.

**Table 1:** Accuracy of syringe infusion pump devices in delivering a prespecified infusion flow rate set at 10 mL/h depending on altitude using weight measurement method [Method A])*

	Syringe infusion pump device				
	DPS Orchestra®	Agilia Injectomat®	Micrel Micropump™ 1	Micrel Micropump™ 2	Micrel Micropump™ 3
Altitude 300 m					
Median	9.78	9.78	9.78	9.72	9.70
(IQR)	(9.57–9.88)	(9.62–9.84)	(9.62–9.88)	(9.43–9.79)	(9.43–9.79)
Min–Max	9.13–9.97	9.02–10.04	8.57–10.04	8.48–9.96	8.47–10.10
Altitude 1700 m					
Median	9.66	9.76	9.69	9.66	9.80
(IQR)	(9.54–9.75)	(9.62–9.85)	(9.45–9.81)	(9.36–9.80)	(9.62–9.90)
Min–Max	8.64–9.92	9.38–10.01	8.17–9.94	9.20–9.92	7.49–10.33

*IQR* Inter-Quartile Range. Median infusion flow rates (*p* = 0.83) and interquartile ranges (*p* = 0.27) did not differ across syringe infusion pump devices. Median infusion flow rates (*p* = 0.15) and interquartile ranges (*p* = 0.71) did not differ depending on altitude. Median infusion flow rates (*p* for interaction =0.32) and interquartile ranges (*p* for interaction =0.66) for syringe infusion pumps did not vary significantly with altitude

At both 300 m and 1700 m, and for all devices, the median infusion flow rate was lower than 10 ml/h. Median flow-rate lay within the variability range required by the manufacturers for the three Micrel devices (±4%, > 9.6 ml/h), and for the Agilia Injectomat® (±3%, > 9.7 ml/h).

At 1700 m only, median flow was outside the specified variability range (±3%, > 9.7 ml/h) for the DPS Orchestra® (9.66 ml/h).

**Fig. 2:**
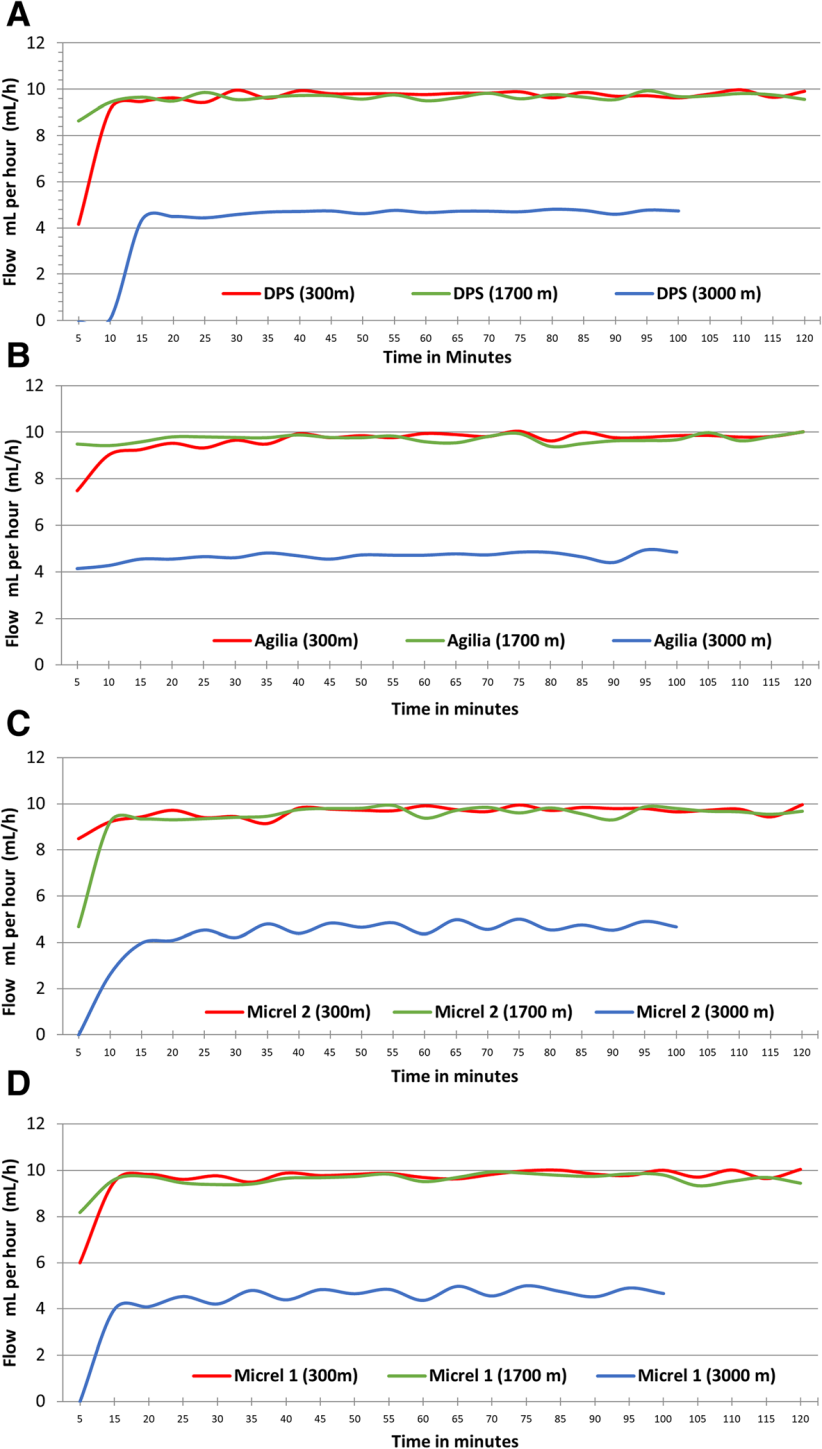
Infusion profiles measured using method A depending on SIP models. **A**: DPS Orchestra®; **b**: Agilia Injectomat®; **c**: Micrel Micropump 1; **d**: Micrel Micropump 2

At 3000 m, due to poor weather conditions that prevented the investigation team from descending to 1700 m before the end of the experiment, only 20 measurements were taken at a prespecified flow rate set at 5 mL/h instead of 10 mL/h by mistake. All devices yielded a median flow lower than the target flow: Micrel Micropump™ 1 [4.54 (4.29–4.81)], Micrel Micropump™ 2 [4.55 (4.30–4.82)], Agilia Injectomat® [4.70 (4.54–4.80)] and DPS Orchestra® [4.68 (4.53–4.73)]. All of the devices tested needed at least 10 min to reach target flow at all altitudes, as shown in Fig. 2.

For a prespecified flow rate set at 5 mL/h (Table 2), the automatic flow measurement method (method B) yielded a significant first-order interaction between devices and altitude levels for the median flow rate (*P* for interaction < 0.001) and interquartile range (*P* <  0.001). While the accuracy of the Agilia Injectomat® and DPS Orchestra® devices was comparable at 300 and 1700 m of altitude, altitude change was associated with decreasing median flow rates and increasing interquartile ranges for the two Micrel Micropump™ devices. Low median flow-rate were observed with the two Micrel Micropump™ both at 300 m (4.4 and 4.0 mL/h) and 1700 m altitude (3.40 and 3.80 mL/h).

**Table 2:** Accuracy of syringe infusion pump devices in delivering a prespecified infusion flow rate set at 5 mL/h depending on altitude using automatic instantaneous flow measurement method [Method B])^a^

	Syringe infusion pump device			
	DPS Orchestra®	Agilia Injectomat®	Micrel Micropump™ 1	Micrel Micropump™ 2
Altitude 300 m				
Median	4.93	5.00	4.40	4.00
(IQR)	(4.66–5.16)	(4.74–5.25)	(3.73–5.49)	(2.91–5.79)
Min–Max	3.59–5.89	3.68–5.96	2.41–14.31	1.66–19.35
Altitude 1700 m				
Median	5.00	4.90	3.40	3.80
(IQR)	(4.84–5.08)	(4.80–4.96)	(2.59–5.90)	(2.55–6.78)
Min–Max	4.05–5.42	4.04–5.16	1.23–12.68	1.27–15.85

^a^ Data were collected using a high-precision flowmeter (Method B; see Methods). A total of 100 measurements per second were collected and results were calculated during the 2nd h in a random sample of 1200 values obtained at steady state. *IQR* Inter-Quartile Range

**Fig. 3:**
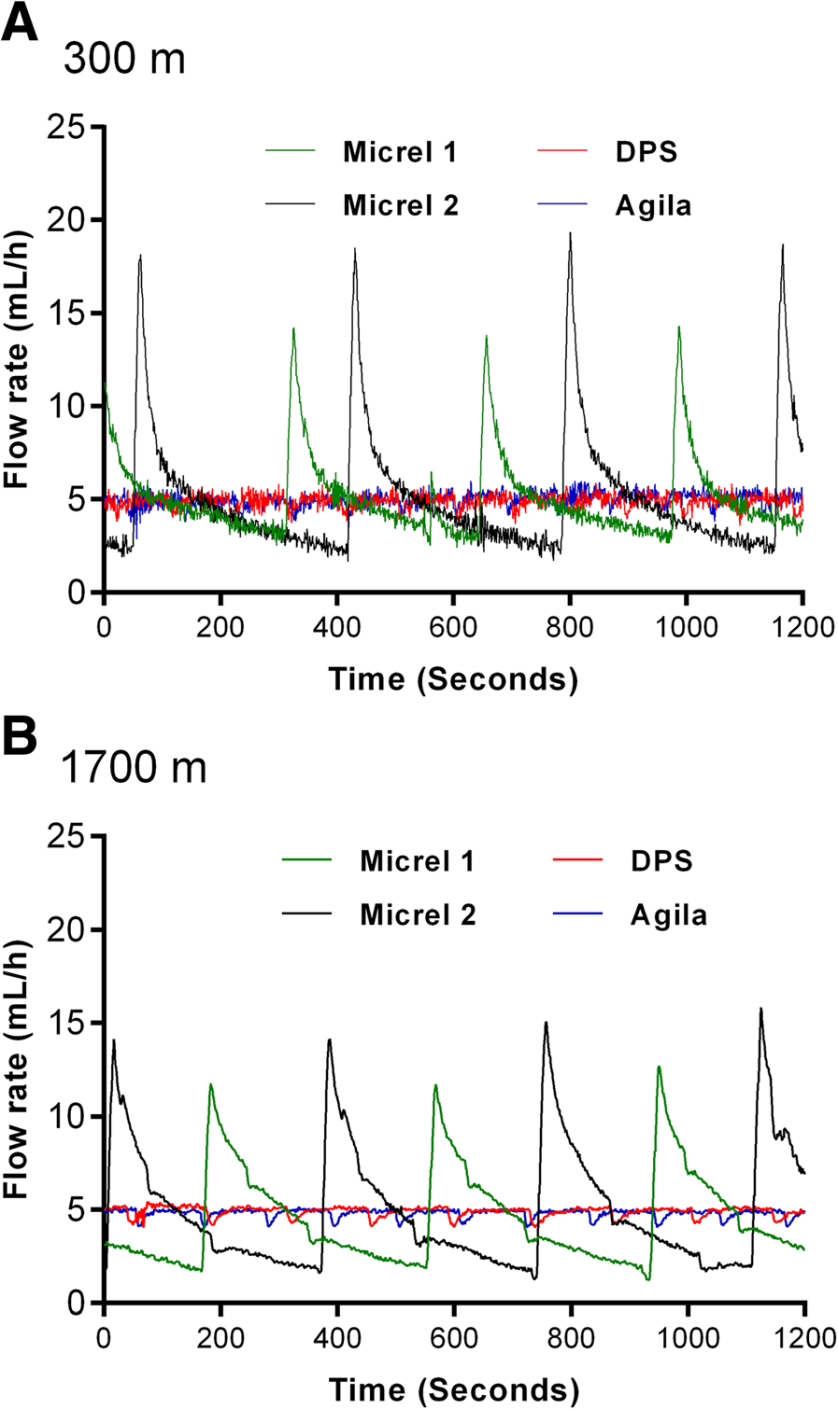
Infusion profile measured using method B (instantaneous flow, CORI-FLOW™ flow meter) at 300 m (**a**) and 1700 m (**b**) altitude, set at 5 ml/h

Method B allowed us to obtain the instant flow-rate profile delivered by each device. This is graphically displayed over a period of 20 min (1200 s at steady state) in the Fig. 3. The Micrel devices provided sequential boluses (from 1.7 to 19.3 mL/h at 300 m and 1.2 to 15.9 at 1700 m) instead of a constant flow of 5 mL/h as provided by the Agilia Injectomat® and DPS Orchestra®.

Starting time, cycle characteristics, and finally the boluses delivered after each mechanical push (calculated area under the curve above the 5-mL/h line) are presented in Table 3.

**Table 3:** Practical characteristics of syringe pump detected by method B (automatic instantaneous flow measurement)

	Starting time (min)	Cycle time (s)	Exceeding the set (%)	Estimated bolus (mL)
DPS Orchestra®				
300 m	0.98	9	20	0.003
1700 m	0.98	14.1	5.4	< 0.001
Agilia Injectomat®				
300 m	1.52	8.6	7.9	< 0.001
1700 m	0.43	12.6	4.6	< 0.001
Micrel Micropump™ 1				
300 m	6.23	33.6	184.6	0.03
1700 m	15.28	46	165.4	0.03
Micrel Micropump™ 2				
300 m	3.89	33.1	218.6	0.04
1700 m	14.05	47	197	0.05

*S*tarting time: time to reach the speed of 4.75 mL/h. Cycle time: time between the beginning of a cycle and the beginning of the next one. Represent the mean time between two modifications (increase/decrease/stop/start) of the flow-rate detected by the flowmeter (technical characteristic). Exceeding the set: maximum value above the target set (expressed as a percentage of target set). Bolus volume: the maximum overshoot “exceeding the set” during the infusion

The starting time increased at 1700 m for the Micrel Micropump™ devices (6–15 min and 3.8–14 min). The cycle time detected was higher at the higher altitudes for all devices. Maximum overshoot during infusion was > 30 μL for the Micrel devices and < 3 μL for the Agilia Injectomat® / DPS Orchestra®.

## Discussion

The results of this study seem to indicate that SIP accuracy varies depending on altitude and the measurement method. It also highlights that compliance with IEC 60601-2-24 standards for electrical medical devices [8] may not fit with clinical concerns.

When using the standard (weight) method, all five devices tested complied with the manufacturer’s requirements at 300 m altitude. Delivered flow rates were consistent with programmed flow rates and no significant difference was found depending on device and altitude. Notably, a previous study using the same measurement method reported significant differences between programmed and delivered volume depending on catheter diameter [9].

The second method tested, which consisted in measuring the instantaneous flow rate with a flow meter, was used previously to assess the accuracy of check valves in SIPs [10]. Interestingly, this measurement method showed that Micrel Micropump™ devices provided lower flow-rates at both altitudes than the prespecified target (with an apparent additional bad effect of altitude) and sequential boluses instead of continuous flow.

Although speculative, the potential implications of this observation may include effects on hemodynamic stability, particularly when using vasoactive drugs such as noradrenaline or adrenaline. Indeed, Greau et al. demonstrated that manual changeover of noradrenaline SIPs had a clinical impact on patient hemodynamic status as compared with automatic changeover (modification of ≥20 mmHg). This effect was stronger for high-concentration solutions and low programmed flow. Manual changeover can be considered as a flow interruption followed by a bolus, very close to Micrel Micropump™ device performance. For this reason we consider that the use of these devices may compromise the clinical status of severely injured patients who may require hemodynamic support, particularly when managing acute severe head trauma [11]. However, very light devices may also be better than nothing in particular situations such as mountain rescue operations [12, 13]. When using a low-weight device is the only available option, the recommendation to use a low-concentration solution with a high-speed flow rate in order to minimize the quantity of active drugs delivered at each bolus, is quite reasonable.

These findings also suggest that altitude may impact mechanical compliance and modify delivered flow due to changes in pressure on devices. To our knowledge, this is the first study focusing on the effect of altitude on flow changes in mechanical infusion therapy. In a recent systematic review, Snijder et al. [14] noted mechanical compliance, resistance and dead volume as the three main causes of flow changes. Presence of air bubbles and vertical displacement of the SIP were also identified as causes of flow variability with a possible clinical effect [15, 16]. A comparable study on the effect of altitude on medical devices was conducted to assess the accuracy of ventilation devices in a simulated hypobaric situation [17]. It clearly highlighted the fact that air pressure had to be taken into account when medical devices were to be used at high altitude for patients with acute respiratory distress. In mirror, these questions were asked when using pumps in hyperbaric conditions [18–23]. It underline more significant variations with “low” infusion flow rate [18], material-dependent flow variations (tube compliance [21, 22], syringe type [19]), but also during the compression and decompression phases [19, 21, 23]. These phases can be compared to the periods of ascension and descent in flight, with the risk of formation of bubbles, modifying the flows. This phenomenon has been blamed for commercial flight take-offs bolus in insulin pump carriers [20].

In our experience, the average altitude for rotor-wing transportation is 1700 m, although some inter-hospital transfers may require flying above mountains more than 3000 m high. Because Micrel Micropump™ accuracy was significantly altered at 1700 m, we advocate the use of standard SIP devices during air transportation. Whether flying through valleys at 1700 m rather than passing above high-altitude mountains is a viable option remains unanswered: the potential impact of SIP inaccuracy when used over 3000 m altitude may be balanced by shorter transportation time.

This exploratory study has several potential limitations. First, the weight measurement (method A) was close to the normative method proposed by the manufacturer in compliance with the IEC 60601–2-24 [8] standard but was not identical. Norms require the use of a precision scale and accuracy measurement is calculated from the total volume delivered in the 2nd h of infusion. We decided to perform iterative measures of volumes during the 2 h of drip, so the technical constraints of this method may have resulted in a loss of data (e.g., drops lost when changing measurement tubes). A limited number of available tubes allowed us to take only 24 volume measurements per SIP over the 2 h, which may have effected measurement precision during the study period. In fact, measurements represented an average flow over 5-min periods.

Second, the prespecified infusion rate was set at 10 mL/h for method A (weight) and 5 mL/h for method B. Although unlikely, we cannot exclude that device accuracy differed according to flow infusion rate. In particular, low flow-rates can lead to increased start-up delays [24]. Yet this does not affect the finding regarding the sequential boluses highlighted by automatic flow measurement.

Third, only four devices were evaluated at 3000 m using the weight measurement method (method A), one of the Micrel having failed at 3000 m, and did not turn back on despite changing the batteries. Relatively few devices were evaluated at three altitudes, which may undermine the generalizability of the findings. Furthermore, the flow rate was set at 5 mL/h by mistake, and precluded any comparison with accuracy at 300 and 1700 m.

Finally, measurements were made indoors, in order to control the ambient temperature. But we did not record the precise atmospheric pressure on the measurement days. The pressure variation related to the weather is about +/− 20 hPA outside of extreme events such as hurricanes. On a scale of altitude the weather variations could correspond to changes of less than 100 m to the highest altitude of measurement. We consider this variation not important enough to have changed our results. However, to be certain that the atmospheric pressure was responsible for the observed changes, a hypo / hyperbaric chamber study would be necessary.

## Conclusions

Low-weight SIPs (Micrel Micropump™), provide sequential boluses instead of continuous flow. Even though they are ten times heavier, standard devices appear to be most reliable, even at different altitudes. Only automatic measurement of instantaneous flow showed this very important difference across devices. We believe that existing norms regarding SIP accuracy may not be relevant for use in the prehospital setting. However, manufacturers should provide information on instantaneous flow.

## Data Availability

The datasets used and/or analysed during the current study are available from the corresponding author.

## Abbreviations

FR: Flow rate
HEMS: Helicopter emergency services
SIP: Syringe infusion pump
